# Parvalbumin Promoter Methylation Altered in Major Depressive Disorder

**DOI:** 10.7150/ijms.36131

**Published:** 2019-08-14

**Authors:** Benjamard Thaweethee-Sukjai, Sirijit Suttajit, Samur Thanoi, Caroline F. Dalton, Gavin P. Reynolds, Sutisa Nudmamud-Thanoi

**Affiliations:** 1 Department of Anatomy, Faculty of Medical Science, Naresuan University, Phitsanulok, 65000, Thailand; 2 Centre of Excellence in Medical Biotechnology, Faculty of Medical Science, Naresuan University, Phitsanulok, 65000, Thailand; 3 Department of Psychiatry, Faculty of Medicine, Chiang Mai University, Chiang Mai, 50200, Thailand; 4 Biomolecular Sciences Research Centre, Sheffield Hallam University, Sheffield, S1 1WB, UK

**Keywords:** DNA methylation, major depressive disorder, parvalbumin, pyrosequencing, suicide attempt

## Abstract

**Aims:** To determine the extent of DNA methylation of parvalbumin gene* (PVALB)* promoter in major depressive disorder (MDD) patients with and without suicide attempt in comparison with healthy controls.

**Methods:** The extracted DNA from dried blood spots of MDD patients (n = 92) including non-suicidal MDD and suicidal-MDD subgroups (n = 45 and n = 47, respectively) and age-matched control subjects (n = 95) was used for DNA methylation analysis at four CpG sites in the promoter sequence of *PVALB* by pyrosequencing.

**Results:** The *PVALB* methylation was significantly increased at CpG2 and decreased at CpG4 in the MDD group compared to the control group, while there was no difference between non-suicidal MDD and suicidal-MDD subgroups. A significant inverse correlation of severity of MDD was indicated only for CpG4.

**Conclusion:** This study provides the first evidence of abnormalities of *PVALB* promoter methylation in MDD and its correlation with MDD severity indicating a role for epigenetics in this psychiatric disorder.

## Introduction

Major depressive disorder (MDD) is a severe neuropsychiatric disorder which is a common public health issue for people worldwide. The World Health Organization (WHO) has reported that 300 million people worldwide suffer from MDD 1. Around 800,000 people die from suicide every year 1. Several studies have reported that psychiatric disorders, especially MDD, have been strongly linked to suicide 2-6. Moreover, patients with MDD have an almost 20-fold greater prevalence of suicide attempt than other patients hospitalized with no history of MDD 7.

Recently, the etiology of MDD and suicide remains unclear. Nevertheless, there is strong evidence for neurobiological deficits in the pathophysiology in MDD and suicide. Abnormalities of major neurotransmitter systems (serotonin, catecholamine, γ-aminobutyric acid (GABA) and glutamate) have been widely reported in both MDD patients 8,9 and suicidal subjects 10,11. Epigenetic mechanisms, which can be modified by environmental factors, may play an important role in MDD pathology by genetic alterations in response to susceptibility or development to diseases 12-14. DNA methylation is the most widely studied epigenetic factor, for which the main mechanism is an addition of a methyl group at the cytosine 5' position in cytosine-phosphate-guanine dinucleotide (CpG) sites 14,15. DNA methylation occurring in or near promoter regions influences, and generally represses, gene transcription. Therefore, DNA methylation is a key mechanism that may contribute to the changes in gene expression seen in psychiatric disorders. Chen et al. have recently reviewed the evidence for the involvement of DNA methylation in depression, demonstrating particularly how brain-derived neurotrophic factor (BDNF) and solute carrier family 6 member 4 (SLC6A4) genes are implicated and that changes in methylation in several other genes emerging from whole-genome methylation studies have been reported 13.

GABA is the most important and abundant inhibitory neurotransmitter in the central nervous system. Recently, psychiatric disorders have been linked with abnormalities of GABAergic neurotransmission in schizophrenia, bipolar disorder and MDD. In MDD, there have been multiple reports of altered GABA content in plasma, cerebrospinal fluid (CSF) and brains of depressed patients as well as changes in GABA-related gene and protein expression 9,16-18. Moreover, altered mRNA GABAA receptor subunit expression was found in postmortem frontal cortex in depressed suicide victims 19. Postmortem microarray studies in a cohort of suicides reported that GABA-related gene expression was altered in suicides both with and without depression in prefrontal cortex 20, limbic system 21 and also global brain 22. Moreover, an association of depression with GABA-related genes indicated by differences in gene expression between suicides with and without depression has been reported 20-22. These findings indicate that GABAergic neurotransmission is likely to be dysregulated in depression and suicide.

GABAergic interneurons can be subdivided into three types based on the expression of the calcium-binding proteins calbindin, calretinin and parvalbumin (PV) 23. Specific deficits of PV-immunoreactive cells are found in the brain in both schizophrenia and its animal models, and recently an increase of PV gene (*PVALB*) promoter methylation has been recently reported in hippocampal tissue from both schizophrenia 24 and its sub-chronic phencyclidine rat model 25. Further evidence implicating *PVALB* promoter hypermethylation in psychosis comes from the study of methamphetamine-induced psychosis subjects 26. While frontal cortical PV protein or *PVALB* expression are not reportedly changed in MDD 27-29, deficits of *PVALB* expression in anterior cingulate cortex have been observed 30. Thus, as there are reported changes in genes and proteins related to GABAergic neurotransmission in MDD, the present study hypothesized that DNA methylation of *PVALB* may be abnormal in this disorder. This study examined DNA methylation within the promoter sequence of *PVALB* in MDD patients both with and without a history of attempted suicide. Exploration of the relationship between DNA methylation in the *PVALB* promoter with symptom severity of MDD was a secondary objective of this study.

## Materials and methods

### Subjects

The subject groups collected for this study comprised 100 unmedicated MDD patients and 100 age- and sex-matched control subjects (18-65 years old). MDD patients were diagnosed by a researcher psychiatrist in Clinical Psychiatry, Maharaj Nakorn Chiang Mai Hospital, Thailand based on criteria in the Diagnostic and Statistical Manual of Mental Disorders, fifth edition (DSM-5). Exclusion criteria for patients included a previous history of schizophrenia, bipolar disorder, or drug abuse. The subjects in the MDD group were divided into two subgroups of non-suicidal MDD and suicidal-MDD groups based on their suicide attempt history determined by the psychiatrist according to the Mini International Neuropsychiatric Interview (M.I.N.I.) (Thai version 5.0.0 - Revised 2007). The 17 Item Hamilton Depression Rating Scale Thai version (HAMD-7) was used for assessing severity of depression. The control subjects completed the Thai Mental Health Indicator (TMHI-66) for evaluation of mental health. Volunteers with previous history of schizophrenia, mood disorder, anxiety, or drug addiction and abnormal mental health were excluded from this study.

The experimental procedures of this study were approved by the Naresuan University Institutional Review Board, Thailand and the Research Ethics Committee, Faculty of Medicine, Chiang Mai University, Thailand. Informed consent forms were obtained from the participants involved.

### Blood sample collection and DNA extraction

Fingertip blood samples were collected on Whatman FTA™ cards (GE Healthcare) using Chelex^®^ 100 resin (Bio-Rad) following a modified protocol from application note 28-9822-22 AA by GE Healthcare (GE Healthcare). Then FTA discs were punched from dried blood spots using a Harris Micro Punch 2.0 mm (GE Healthcare, USA) and used for DNA extraction. Briefly, FTA discs were washed three times in 1 ml high-purity water by incubation at room temperature for 10 min with occasional inverting, centrifugation at 20,000 x g for 3 min and discarding the water in each time. Subsequently, 5% Chelex^®^ 100 resin (Bio-Rad) was added and incubated at 56°C for 20 min. The samples were mixed by vortexing, further incubated at 100°C for 8 min and then centrifuged at 20,000 x g for 3 min. The supernatant containing genomic DNA was carefully collected and the concentration determined using NanoDrop^®^ ND-1000 (Thermo Fisher Scientific).

### Bisulfite conversion and methylation analysis

Bisulfite conversion of 500 ng genomic DNA template for each reaction was performed using EpiTect^®^ Fast DNA Bisulfite Conversion kit (QIAGEN) according to the manufacturer's protocol.

**Figure 1:**
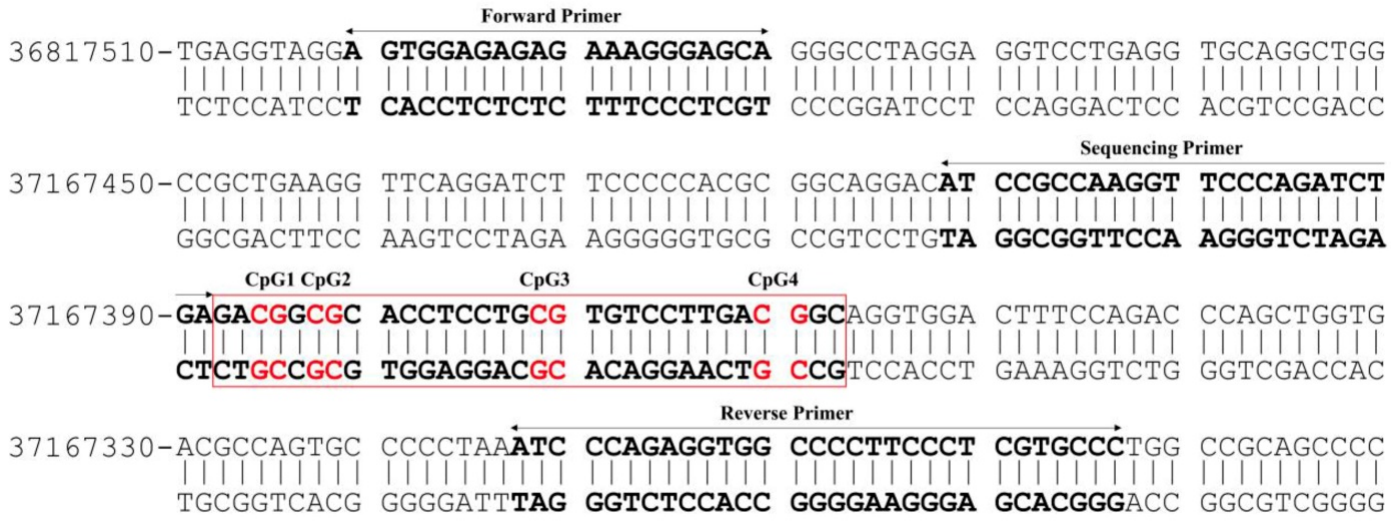
Illustration of the CpG sites and primers used for DNA methylation analysis of *PVALB* promotor region on chromosome 22. The target region consists of 217 bp containing four CpG sites.

The extent of DNA methylation within a promoter sequence of *PVALB* (Homo sapiens chromosome 22, GRCh38.p12 Primary Assembly, NCBI Reference Sequence: NC_000022.11 (36800701..36819473, complement)) (Figure 1) and long interspersed nucleotide element-1 (*LINE-1*) genes was evaluated using the pyrosequencing technique according to the protocol recommended by the manufacturer (QIAGEN). The present study used a set of primers: forward primer: 5'-AGTGGAGAGAGAAAGGGAGTA-3', biotinylated reverse primer: 5'-AACACCAAAAAAAAAACCACCTCTAAAAT-3' and sequencing primer: 5'-ATTAGTTAAGGTTTTTAGATTTGA-3' (Eurofins MWG Operon) 24,26 to quantify methylation of four CpG sites of *PVALB.* In addition, predesigned PyroMark^®^ CpG *LINE-1* Assay (QIAGEN) containing proprietary forward, biotinylated reverse and sequencing primers to detect three CpG sites located at positions 331 to 318 of *LINE-1* (GenBank accession number X58075) was used.

**Figure 2:**
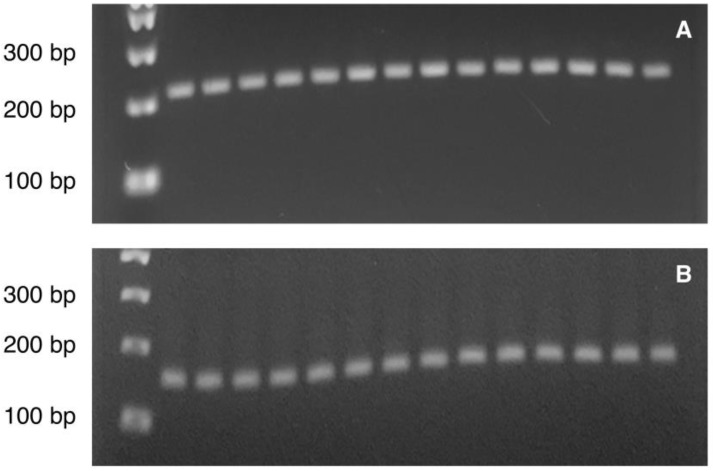
Illustration of the PCR product bands of (A) *PVALB* (217 bp) and (B) *LINE-1* (146 bp) on agarose gel visualized under ultraviolet (UV) illumination. Lane 1: 100 bp DNA ladder**.** Lane 2-8: Control sample 1-7**.** Lane 9-15: MDD sample 1-7.

The PCR reaction included 1x PyroMark PCR Master Mix, 1x CoralLoad Concentrate (only for the reaction of *PVALB*), 0.2 µM of forward and reverse primers, RNase-free water and 1 ng bisulfite-converted DNA template in 25 µl PCR final volume. The PCR amplification was conducted with initial PCR activation step at 95°C for 15 min, 45 cycles of denaturation at 94°C for 30 s, annealing at 56°C (for *PVALB*) / 50°C (for *LINE-1*) for 30 s and extension at 72°C for 30 s, and final extension step at 72°C for 10 min. A quality of PCR product was checked by agarose gel analysis with ethidium staining prior to Pyrosequencing analysis (Figure 2A-2B). Then, ten microliter of PCR product was immobilized with Streptavidin Sepharose^TM^ High Performance (GE Healthcare) and purified using the PyroMark Q24 Vacuum workstation following the manufacturer's instructions (QIAGEN). The samples then underwent pyrosequencing to determine methylation using the PyroMark Q24 system according to the instructions from the manufacturer (QIAGEN). The percentage methylation at each CpG site in a pyrogram was calculated by Pyromark^®^ Q24 2.0.6.20 software (QIAGEN). All results which were labelled as “check” or “failed” were repeated from the PCR amplification step. All samples that failed to provide reliable results after replication were rejected.

### Statistical analysis

The Statistical Package for the Social Sciences (SPSS) software version 17.0 for Windows (SPSS Inc., Chicago, IL, USA) was used for statistical analysis. A difference in sex distribution was tested using Chi-square tests. Comparisons of differences in age, duration of illness, HAMD-17 score and percentage of DNA methylation were analyzed using non-parametric Mann-Whitney test or Kruskal-Wallis test. Correlation was evaluated using Spearman's rank correlation. The significance level was set at p < 0.05. GraphPad Prism^®^ version 8.0 for Windows (La Jolla, CA, USA) was used to create scatter dot plots.

## Results

**Figure 3:**
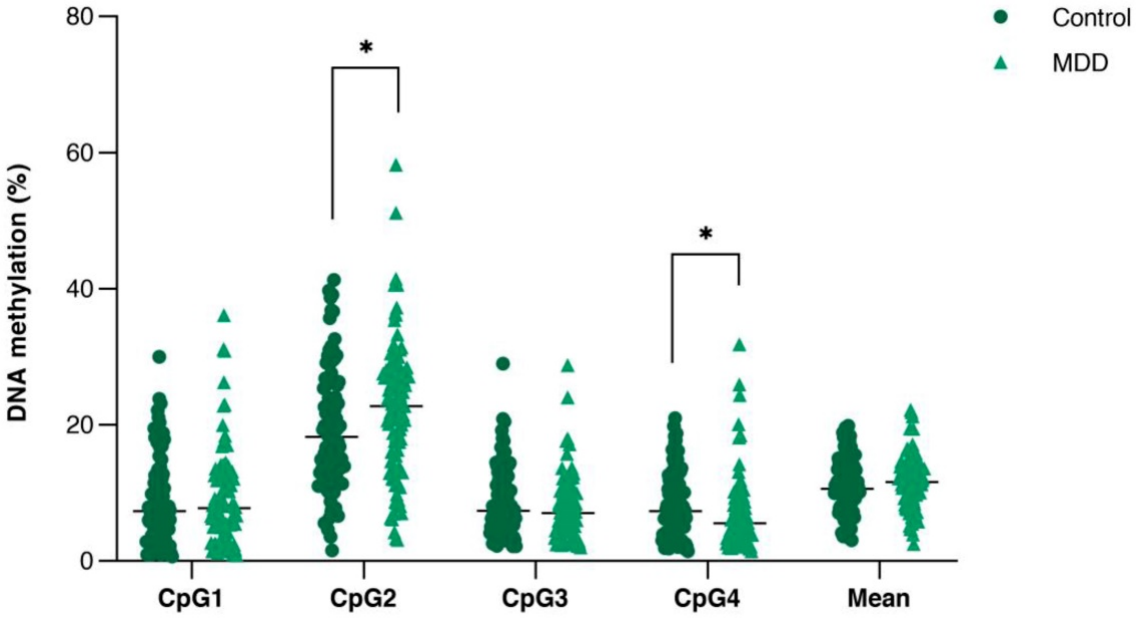
Comparison of DNA methylation (%) of the four consecutive CpG sites in *PVALB* promoter between control and MDD groups. Scatter dot plots of each groups are horizontally lined with the median value. * p < 0.05. MDD: Major depressive disorder.

**Figure 4:**
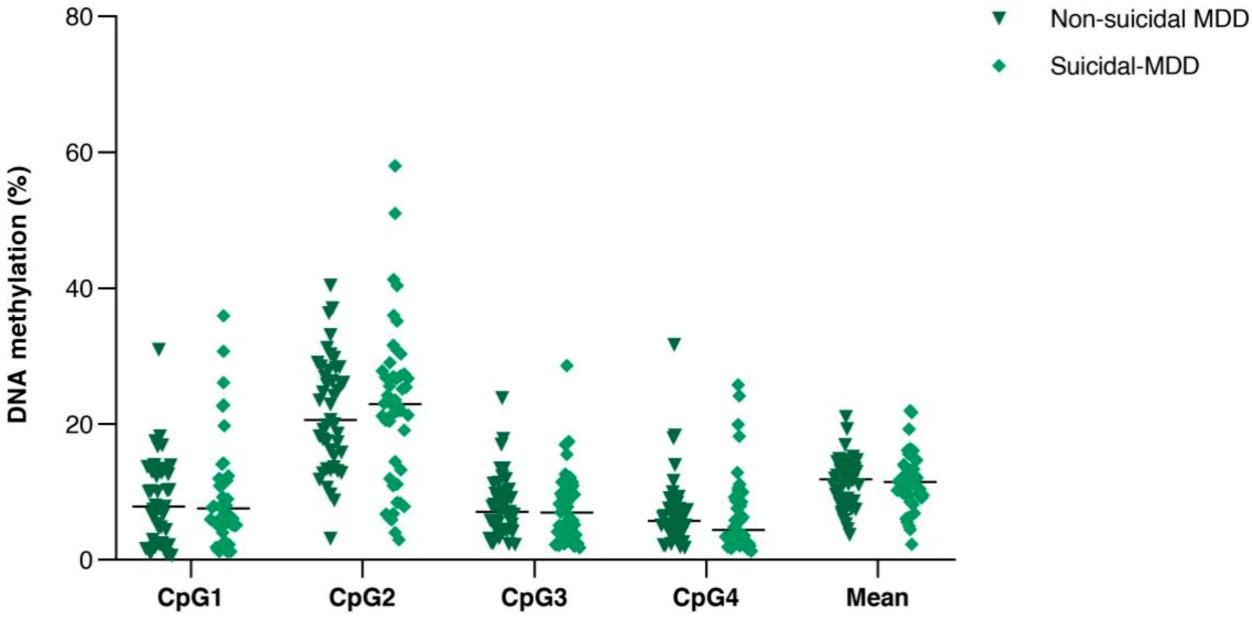
Comparison of DNA methylation (%) of the four consecutive CpG sites in *PVALB* promoter between non-suicidal MDD and suicidal-MDD groups. Scatter dot plots of each groups are horizontally lined with the median value. MDD: Major depressive disorder.

All samples showed single PCR bands without evidence of DNA degradation before determination of *PVALB* and* LINE-1* methylations (Figure 2). Eight MDD and five control samples were rejected on the basis of unreliable pyrosequencing results for either *PVALB* or *LINE-1*, leaving an MDD group of 68 females and 24 males (43.13 ± 14.72 years old) and a control group of 71 females and 24 males (43.06 ± 15.03 years old) with complete data. DNA methylation status of the four CpG sites in the *PVALB* promoter sequence of MDD and control groups is shown in Figure 3. While the mean percentage methylation at the four CpG sites showed no significant effect in MDD group (Z = -0.762; p = 0.446), a significant increase in percentage methylation at CpG2 (Z = -2.021; p = 0.043) and a significant decrease in percentage methylation at CpG4 (Z = -2.161; p = 0.031) were seen. The methylation at each of these two CpG sites was not significantly different when comparing non-suicidal MDD and suicidal-MDD groups (Z = -0.144; p = 0.885 and Z = -1.172; p = 0.241, respectively) (Figure 4), nor between these MDD subgroups and control group (ꭓ^2^ = 4.090; df = 2; p = 0.129 and ꭓ^2^ = 5.571; df = 2; p = 0.062, respectively).

Mean *LINE-1* methylation showed no significant difference between MDD and control groups (70.85 ± 1.63 vs 70.95 ± 1.68; Z = -0.389; p = 0.697), nor between non-suicidal MDD and suicidal-MDD groups (70.77 ± 1.33 vs 70.93 ± 1.89; Z = -0.894; p = 0.371). Methylation of CpG2, but no other *PVALB* sites, was significantly negatively correlated with *LINE-1* methylation in the control group (r = -0.230, p = 0.025), but not in the MDD group (r = -0.184, p = 0.079).

**Figure 5:**
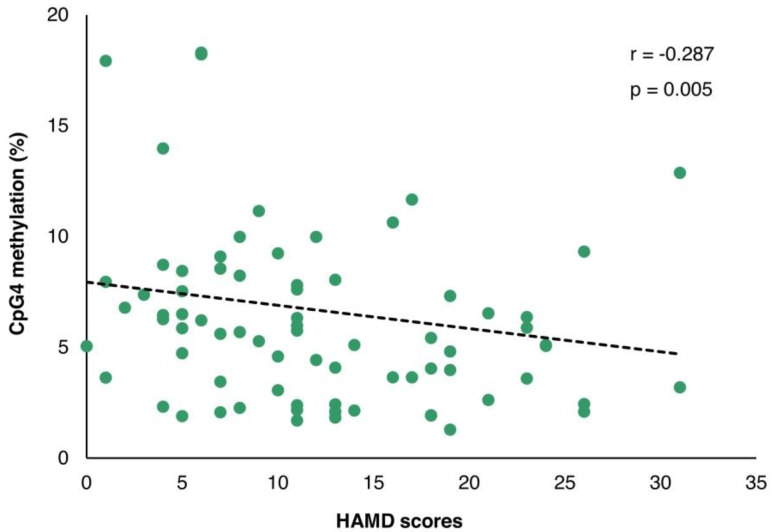
The correlation of *PVALB* methylation at CpG4 and HAMD-17 scores among MDD patients. HAMD: Hamilton depression rating scale.

The demographic and clinical variables are shown in Table 1. A significant correlation between age and *LINE-1* methylation was observed in both control and MDD groups (r = -0.331; p = 0.001 and r = 0.328; p = 0.001, respectively) with no difference in age neither between control and MDD groups nor between non-suicidal MDD and suicidal-MDD subgroups (data not shown). The duration of illness in the non-suicidal MDD group was significantly lower than the suicidal-MDD group (Z = -2.534; p = 0.011). Moreover, a significant correlation of methylation with duration of illness was found for *LINE-1* (r = 0.266; p = 0.010), but not for any *PVALB* site (data not shown). Although no significant difference of HAMD-17 score was observed between non-suicidal MDD and suicidal-MDD groups (Z = -1.212; p = 0.225), a highly significant correlation with HAMD-17 score was apparent for CpG4 methylation (r = -0.287; p = 0.005), but not for CpG2 (r = -0.024; p = 0.820) (Figure 5). This effect remained after controlling for age and sex in a partial Pearson correlation analysis (p = 0.016).

## Discussion

The main finding in this study was the site-specific change in *PVALB* promoter methylation which was increased at CpG2 and decreased at CpG4 in Thai MDD patients. This occurred along with no change of global DNA methylation represented by *LINE-1* methylation. Moreover, we found a strong association of *PVALB* promoter methylation at CpG4 with the severity of depression, but no association with suicide attempt in MDD patients.

The present study has assessed DNA methylation at the same CpG sites of the *PVALB* promoter as previous studies in humans 24,26, and (for CpG2) the equivalent sequence in rats 25. Interestingly, the finding of elevated DNA methylation at CpG2 was also previously observed associated with schizophrenia 24 and methamphetamine dependence, especially methamphetamine-induced psychosis 26, in humans, and at the equivalent site in brain tissue from rats receiving phencyclidine 25. While the previous consistent findings indicate that hypermethylation of *PVALB* at CpG2 might be generally associated with psychotic illness and animal model of schizophrenia, these results suggest that this epigenetic change may extend to other psychiatric disorders, in this case MDD.

The decrease in methylation at CpG4 associated with MDD is in contrast to the increase at this site in post-mortem hippocampus in schizophrenia subjects 24, while no significant difference was identified in methamphetamine-induced psychosis subjects 26. The validity of this finding is supported by the highly significant inverse correlation of CpG4 methylation with the severity of depression indicated by HAMD-17 scores, an independent effect of age or sex. Reanalysis of the data excluding outlying subjects gave essentially the same result (data not shown).

In the mammalian genome, most CpG sites are normally methylated while CpG residues enriched in CpG islands, particularly located in or near gene promoter regions, are relatively protected from methylation. An elevation of DNA methylation or hypermethylation at such CpG islands often results in blocking transcriptional initiation leading to repression of gene expression 31, while a reduction of DNA methylation or hypomethylation may lead to increased gene expression 32.

However, the direct relationship between DNA methylation and gene expression of *PVALB* is unknown, although the findings described above in schizophrenia and its animal model show both increased methylation and reduced expression of *PVALB*. In contrast, previous studies in postmortem brain in MDD have demonstrated no changes of *PVALB* gene expression or neuronal immunoreactivity in most brain regions studied 27-29,33, other than the anterior cingulate 30. These findings imply that the *PVALB* methylation profiles might influence the pathology of MDD in specific brain regions. To support this hypothesis, hypermethylation of the *PVALB* promoter has been identified specifically in only hippocampus, but not prefrontal cortex, in schizophrenia 24.

*LINE-1* methylation provides an indication of global DNA methylation levels. This study has found that the *LINE-1* methylation was positively correlated with age, but not associated with MDD. A positive relationship between *LINE-1* methylation and age is well established and has also been reported in psychiatric disorders 34. However, unchanged *LINE-1* methylation from this study is in agreement with a previous finding in blood samples in MDD 35. The lack of any indication of a change in global DNA methylation indicates that the findings in *PVALB* are independent of such an effect and perhaps specific for that gene.

One important but inevitable limitation of this study is that the *PVALB* promoter methylation was determined in DNA derived from peripheral blood samples. While studies in post-mortem tissue from psychiatric patients have been undertaken 24, DNA methylation is most often investigated in blood samples, while there is evidence from previous studies of concordant changes in methylation between blood and brain 36. We have also observed increased *PVALB* methylation in blood samples similar to reported changes in brain tissue in schizophrenia (Reynolds et al., unpublished data). There have been other findings of DNA methylation from blood samples of patients with MDD. Changes in methylation profiles of specific genes such as zinc finger and BTB domain containing 20 (*ZBTBT20*) 37, *BDNF* 38-42 and nuclear receptor subfamily 3, group C, member 1 (*NR3C1*) 43 have been reported. We do not know the direction of causality in these findings, i.e. whether the changes in PVALB methylation are a contributor to, or a consequence of, MDD and, for CpG4, depressive symptom severity. While some of these findings may also be confounded by drug treatment, we can exclude this influence this influence in the current study using unmedicated subjects.

In summary, this study provides the first evidence of changes in the *PVALB* promoter methylation in MDD. *PVALB* promoter methylation was increased at CpG2 and decreased at CpG4 in MDD patients, effects that were independent of a measure of global DNA methylation. Moreover, the decrease in *PVALB* methylation at CpG4 correlated strongly with severity of depression. Further studies need to determine how these findings might reflect changes in *PVALB* methylation within the brain and their impact on gene expression and, eventually, GABAergic neuronal function.

## Figures and Tables

**Table 1:** Demographic data and clinical characteristics of control and MDD groups, and non-suicidal MDD and suicidal-MDD subgroups.

	Control (n = 95) (mean ± SD)	MDD (n = 92) (mean ± SD)	p-value	Non-suicidal MDD (n = 45) (mean ± SD)	Suicidal-MDD (n = 47) (mean ± SD)	p-value
**Age (years)**	43.06 ± 15.03	43.13 ± 14.72	0.928	42.62 ± 15.15	43.62 ± 14.45	0.860
**Sex (n, female/male)**	71/24	68/24	0.897	35/10	33/14	0.409
**Duration of illness (years)**				5.30 ± 5.75	10.15 ± 10.25	0.011*
**HAMD-17 (scores)**				11.69 ± 7.43	13.47 ± 7.38	0.225

Data are represented as mean ± SD or number (n). * p < 0.05. HAMD: Hamilton depression rating scale; MDD: Major depressive disorder.
